# Prediction method of coal mine gas occurrence law based on multi-source data fusion

**DOI:** 10.1016/j.heliyon.2023.e17117

**Published:** 2023-06-09

**Authors:** Huice Jiao, Weihua Song, Peng Cao, Dengming Jiao

**Affiliations:** a College of Mining, Liaoning Technical University, Fuxin, 123000, China; b College of Safety Science and Engineering, Liaoning Technical University, Huludao, 125105, China; c College of Safety Science and Engineering, Liaoning Technical University, Fuxin, 123000, China

**Keywords:** Multi-source data fusion, Coal mine gas storage, Law prediction, Optimal value of weighting factor

## Abstract

To improve the prediction accuracy and time-consuming of coal mine gas occurrence law (OL), a new prediction method based on multi-source data fusion is proposed in this paper. Firstly, the method obtains the data of coal mine gas OL, determines the key data required in prediction through decision matrix, and preprocesses the data to reduce the influence of regular noise data. This paper analyzes the basic principle of multi-source data fusion, constructs the prediction model of coal mine gas OL with this technology, takes the optimal value of weighting factor as the input value of the model, and completes the design of coal mine gas OL prediction method based on multi-source data fusion. The experimental results show that the accuracy of this method can reach 98%, while that of the other two traditional methods is lower than the existing methods. This method has high accuracy and efficiency in predicting the coal mine gas OL.

## 1 Introduction

Coal is the most important primary energy in China, bearing the historical task of economic development, social progress and national rejuvenation. At the same time, coal is the blood of modern industry, and its safety is the top priority of national industrial safety work [1]. China is rich in coal resources, with coal production and consumption ranking among the top in the world, accounting for a large proportion in the composition of China's energy consumption. Coal consumption will account for 56.0% of the total consumption of primary energy in 2021. Therefore, coal will remain one of the main energy sources in China for a long time [2,3]. Affected by the global energy supply situation in recent years, the coal price has risen year by year, the production pace of coal enterprises has been further accelerated, and coal security has gradually become the focus of researchers. Gas (coalbed methane) is a gas geological body related to coal seams. It is not only a green energy, but also the main driving force of gas outburst and an important factor affecting coal mine safety. The occurrence and migration of gas is restricted and affected by many geological conditions, such as tectonic evolution, coal reservoir structure, burial depth, stress, hydrology and overburden conditions. Revealing and predicting the geological laws of coal mine gas has important guiding significance for coal mining and exploration and prediction of coal seam gas rich areas. The gas discharge prediction is based on the actual location conditions, mining scale and mining technology, using the measured data and historical gas data of the mine to predict the gas discharge, and fully considering various influencing factors such as the gas weathering zone and gas in the coal seam. The predicted data can provide a reasonable theoretical basis for mine ventilation mode, coal face layout, coal mine panel and even the whole coal mine design. Therefore, studying the distribution law of coal seam gas and predicting the discharge amount of coal seam gas are the top priorities for safe and efficient production of high gas mines [4,5].

In recent years, many researchers have attached importance to the prediction of gas OL, and have made some phased achievements. Among them, many algorithms, such as gas geological unit division and evaluation, three-level gas geological map compilation, fuzzy mathematics, neural network algorithm, fractal, multi factor analysis methods, have been successfully applied in the prediction of gas geological laws, and have effectively guided the coal mine and coal bed gas production practice [6]. However, the current method is still difficult to characterize the difference of gas occurrence in local areas. There is a big difference between actual and virtual situations by only relying on experience and a single mathematical model to describe the gas geological law, that is, the accuracy of the prediction method is not high, and it is difficult to meet the actual demand, which needs further exploration and improvement [7]. The multi-source data fusion technology integrates all the information obtained from the survey and analysis, evaluates the information uniformly, and finally obtains unified information, which are widely used in geological and mineral work and regional geological and mineral survey. Therefore, a prediction method of coal mine gas OL based on multi-source data fusion was proposed. First, the key data needed in the prediction is determined by the decision matrix, and then the data is preprocessed on this basis to reduce the impact of conventional noise data. Then the basic principle of multi-source data fusion is analyzed, a prediction model of coal mine gas OL based that is constructed, and the optimal value of the weighting factor is taken as the input value of the model, so as to complete the prediction design.

### 1.1 Data extraction and preprocessing of coal mine gas occurrence

#### 1.1.1 Data extraction of coal mine gas occurrence

Coal seam gas data is its gas content, which is defined as follows: it refers to the amount of gas contained in coal per unit volume or weight. It is not only the basis of calculating gas reserves and predicting gas emission, but also one of the critical parameters to determine the risk of coal and gas outburst. The determination methods include direct and indirect methods. The direct determination method is to extract gas directly from coal and rock, and then determine the composition and content of the extracted sample. In contrast, the indirect determination method is to first determine the porosity, and values of coal and rock, then conduct industrial analysis of coal and rock, and finally calculate the gas content. In contrast, the direct determination method is quick and simple, so it is selected to determine the coal seam gas content in this determination. It can only be used when the analytical instrument is extremely airtight and the sampling site meets the requirements. Its error value is larger than that of the indirect determination method, and it can only be used in smaller measurement. The direct method to determine the gas content of coal seam is to drill the coal seam, remove the original coal drill cuttings sample, determine the gas desorption amount of the mined coal seam by desorption hair, and directly determine the gas desorption amount of coal seam by desorption method. The analytical determination principle is: the measured gas desorption amount of the coal sample is fitted with the desorption law calculated from the gas desorption amount. It aims to calculate the amount of gas lost in the passage of time from the initial time of coal sample collection to the time before tank desorption determination, and then use the residual gas in the coal sample after desorption determination. Through these two steps, the coal seam gas content is jointly deduced [8,9].

There is a certain amount of loss in the determination of coal seam gas content. Therefore, it is necessary to calculate the volume of loss under standard pressure. The study calculates the loss under standard pressure through the direct measurement method of coal seam gas content, as shown in equation (1).

(1) $$T_{i}=\frac{273.2\left(a_{0}-9.81h_{w}-a_{s}\right)T}{1.0313\times 10^{5}\left(273+c_{W}\right)}$$

Among them, $T_{i}$ is the desorption gas content in coal; T means the absorption gas measurement; $a_{0}$ indicates the standard air pressure; $h_{w}$ expresses the desorption velocity measurement; $c_{W}$ refers to the temperature when measured, and $a_{s}$ indicates the maximum water vapor pressure under the coal seam [10].

The gas content of coal sample can be calculated according to the four data of desorption, residual, lost gas amount of coal samples and weight of combustible substances in coal, as shown in equation (2).

(2) $$H=\left(b_{0}+b_{1}+b_{2}\right)/G_{0}$$

In equation (2), H is the content of combustible gas in measured samples; $b_{0}$ denotes gas desorption volume; $b_{1}$ indicates loss gas volume; $b_{2}$ stands for residual gas volume, and $G_{0}$ represents the mass data of combustible gas.

#### 1.1.2 Preprocessing of coal mine gas occurrence data

Due to the influence of the above two gas migration laws and the gas precipitation law of residual coal in goaf, the gas distribution in goaf has a certain regularity. With the advance of the mining face, the floating coal remaining in the goaf will gradually release gas. At the initial stage of gas release from floating coal, the gas concentration within a certain range from the working face will gradually increase [5]. In the area close to the working face, due to the large gap of caving rock in this area and the large flow velocity of leakage air passing through this area, the gas concentration will gradually decrease. After the gas concentration reaches a stable value, the amount of gas released by the floating coal is very small and can be ignored, so the gas concentration is basically unchanged at this time. Therefore, it is necessary to preprocess and analyze the collected gas data to effectively predict the subsequent OL [11].

In this paper, it needs to determine the decision matrix. The multi-attribute decision problem available in the MA [12], as shown in equation (3).

(3) $$X=\left\{x_{1},x_{2},...x_{m}\right\}$$

In equation (3), $x_{m}$ is key attributes; the attribute values of the Y in the data are expressed as shown in equation (4).

(4) $$Y=\left\{y_{i1},y_{i2},...y_{im}\right\}$$

When the target function is ${y_{i}}_{j}$, $y_{ij}=\left[y_{i1},...y_{in}\right]$. At this time, the key data required in the gas memory rule can be listed as the decision matrix. The key data predicted by the gas memory rule are shown in Table 1:

**Table 1:** Key data decision matrix of gas OL.

	$y_{1}$	…. …	$y_{j}$	…. …	$y_{n}$
$x_{1}$	$y_{11}$	…. …	$y_{1j}$	…. …	$y_{1n}$
…. ….	…. …	…. …	…. …	…. …	
$x_{j}$	$y_{i1}$	…. …	$y_{ij}$	…. …	$y_{in}$
…. ….		…. …	…. …	…. …	
$x_{n}$	$y_{m1}$	…. …	$y_{mj}$	…. …	$y_{mn}$

The determined data is preprocessed according to the key data predicted by the gas allocation rule defined above. The original decision matrix is showed in equation (5).

(5) $$Y=\left\{y_{ij}\right\}$$

The transformed decision matrix [13], is showed in equation (6).

(6) $$Z=\left\{z_{ij}\right\}$$

Setting $y_{j}^{\max}$ is the maximum value and $y_{j}^{\min}$ is the minimum value in the *j* column in the decision matrix, and if *j* represents the benefit attribute, as shown in equation (7).

(7) $$z_{ij}=\frac{y_{ij}}{y_{j}^{\max}}$$

When data preprocessing in the above way, the transformed worst attribute value is not necessarily 0 and the best attribute value is 1 [14]. If the j means a cost attribute, it exists in equation (8).

(8) $$z_{ij}=1-\frac{y_{ij}}{y_{j}^{\max}}$$

In equation (8), the best attribute is now 0. Whether cost or benefit property [15], vector normalization is transformed with the following equation to obtain as shown in equation (9).

(9) $$z_{ij}=\frac{y_{ij}}{\sqrt{\sum_{i=1}^{m}y_{ij}^{2}}}$$

This transformation is linear, and it is different from the previous transformations, which cannot distinguish the attribute value from the size of the transformed one, but its biggest feature is that the sum of square of the unified attribute value of each scheme is one. Sometimes the scheme attribute values of a certain target tend to vary widely, or for some particular reason only one scheme is particularly prominent. If these data are preprocessed in general methods, the role of this property in the evaluation will be improperly inflated. For this purpose, it can use a statistical average method similar to the evaluation method: it sets the mean of the scheme and this attribute in the scheme set X to percentage average *M*, and transform it with the following equation (10).

(10) $$z_{ij}=\frac{y_{ij}-\bar{y_{j}}}{y_{j}^{\max}-\bar{y_{j}}}\left(1-M\right)+M$$

In equation (10), $y_{j}$ represents the results of the data processing, and *M* expresses the number of data.

### 1.2 The prediction method based on multi-source data fusion

#### 1.2.1 Multi-source data fusion

**Fig. 1:**
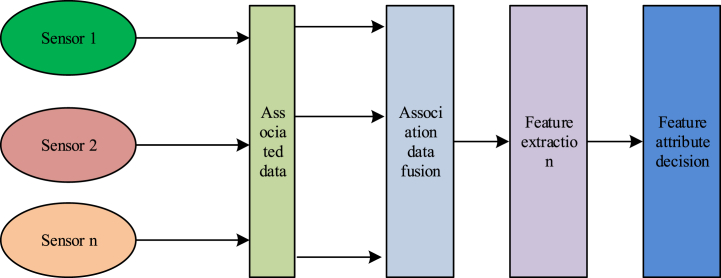
A multi-source data fusion model.

In the multi-source data-level fusion model, the system handles no directly fused data from the same category of sensors, then performs the data feature extraction and completes the attribute judgment of the fusion data, as shown in Fig. 1. Data-level fusion is the lowest one, whose fusion data must be from the same or a similar sensor (e.g., temperature sensing sensor). In addition, to ensure that the fused data come from the same target, the data association operation completes the from the original data [16].

The main advantage of this data-level fusion model is that only a small amount of data is lost and provides relatively much detailed information about the tested target, so the data accuracy tends to be higher. The limitations of data-level fusion are mainly manifested as follows:

(1) Since most of the sensing data to be fused are original data, the amount of data is large, the processing time of the background system is long, and the real-time performance of the system is poor:
(2) Data level fusion is carried out at the bottom of information processing, so the performance of a single sensor node has a direct impact on the results of data level fusion.
(3) Data level fusion is the direct fusion of the original data, so the sensing data must belong to the same type, that is, the fusion model can only provide the fusion of a single attribute of the measured target;
(4) The data traffic is very large, so it has high requirements for the communication environment of the system and poor anti-interference ability.

#### 1.2.2 Design of prediction method of gas allocation rule in coal mine

**Fig. 2:**
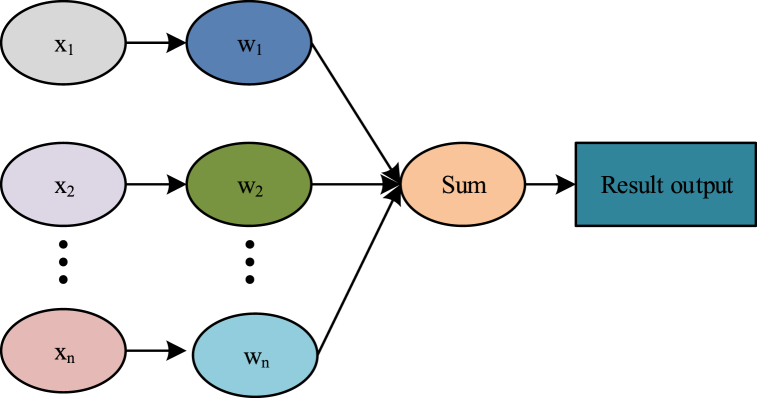
A model of the adaptive fusion algorithm.

According to the above analysis, this paper uses adaptive weighted average to find the corresponding optimal weighted value of each sensor through adaptive search, and to obtain the optimal fusion result if the total mean square error is minimum. The model of the adaptive fusion algorithm is shown in Fig. 2:

The set value of n sensors set in coal mines is $x_{1},x_{2},...,x_{n}$ as the true value to be estimated and the variance is $\sigma_{1}^{2}，\sigma_{2}^{2}...\sigma_{n}^{2}$. They are independent of each other and are unbiased by valuation, with $w_{i}$ representing the corresponding weights, with the fusion of the weighted factors satisfied as shown in equation (11).

(11) $$\overset{˅}{X}=\sum_{i=1}^{n}w_{i}X_{i}$$

Since they are independent from each other, they can be obtained as shown in equation (12).

(12) $$E\left(X-X_{i}\right)\left(X-X_{j}\right)=0,1,2....n$$

The total mean-squared error is in equation (13).

(13) $$\Delta^{2}=E\left[\sum_{i=1}^{n}w_{i}^{2}\left(X-X_{i}\right)\right]$$

Equation (13) is a multivariate quadratic function of a weighted factor that must have a minimum [17]. The polima is used according to the multivariate function. At the hourly total mean square error, the weighted factor is:

(14) $$w_{i}^{*}=\frac{I}{\Delta_{i}^{2}\sum_{i=1}^{n}\frac{1}{\Delta^{2}}}$$

In equation (14), I means source and sink items. According to the determined weighting factor, it is necessary to obtain the optimal solution of the factor [18]. Later, the optimal solution obtained is the data optimal value obtained by the sensor in the coal mine as the key parameter of the prediction model to complete the design of the method. Among them, the optimal solution of the weighted factor can be obtained by equation (15):

(15) $$x_{i}=X+e_{i},x_{j}=X+e_{j}$$

Among them, $e_{i}$, $e_{j}$ stand for different measurement error values and $x_{j}$ represents monitoring values.

A predictive model of the gas assignment rule is constructed according to the determined optimal factors. The gas content of the coal bed is the sum of the gas amount of adsorption and free gas content in the coal bed can be obtained according to the gas equation and the adsorption gas content. The coal gas content X is the sum of the above 2, but the above equation involves many parameters and is inconvenient in actual calculation, so the above equation is further simplified as shown in equation (16).

(16) $$X=x_{i}\frac{ABP\left(100-X\right)}{100\left(1+x_{j}\right)e_{j}}$$

Preventative prediction is constructed based on simplified data, yielding as shown in equation (17).

(17) $$d^{2}\left(X,M\right)=\frac{{\left(X-U\right)}^{\prime }\left(X-U\right)}{{\left(X-U\right)}^{2}}\sigma^{2}$$

**Fig. 3:**
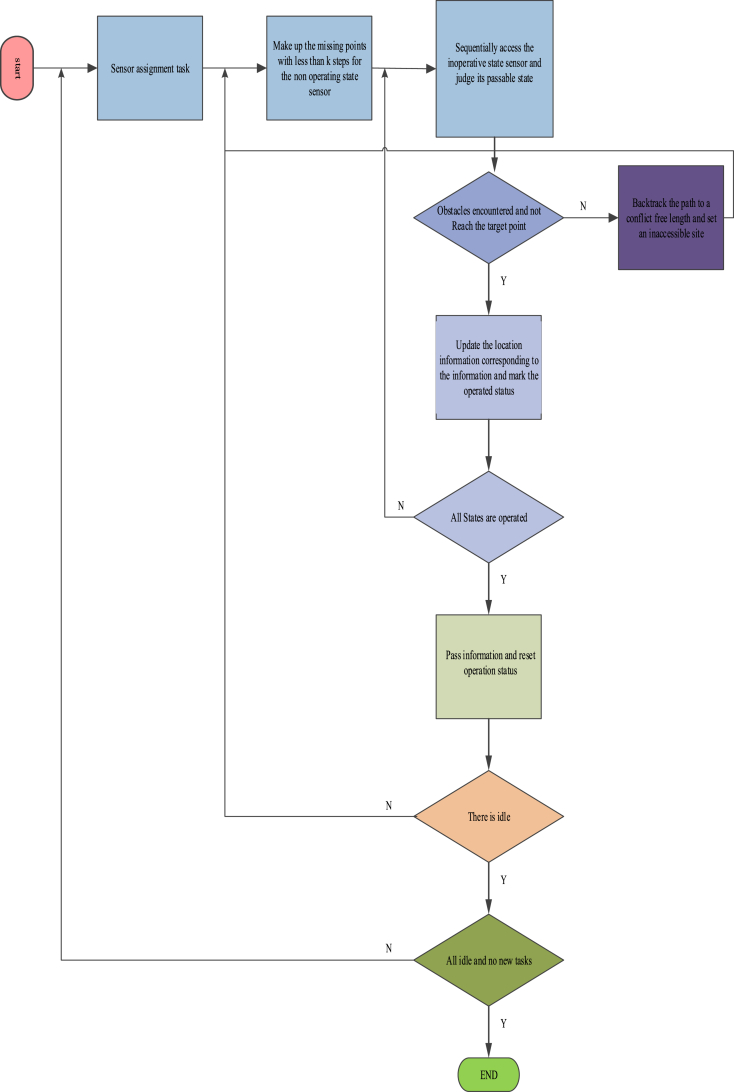
The prediction of coal mine gas storage Laws 4 experimental analyze.

In equation (17), U indicates adsorbed gas content; σ denotes allocation factor. It needs to complete the prediction of the constructed prediction model [19]. The prediction of coal mine gas allocation rule is shown in Fig. 3:

#### 1.2.3 Experimental scheme

In the experiment, a mine field in a geological and mining area was taken as the experimental object. The experimental object was located in the north of the axis of Huainan syncline, the south wing of Panji anticline and the East dip turning end. It was supported between Fengtai fault and ShangtangjiMinglongshan fault from north to south, and cut by F5 and XinchengkouChangfeng fault from east to west. The mine field structure was mainly composed of oblique shear, tension and torsion faults, and the level of compression and torsion faults [20,21]. Tensional and torsional faults could be divided into two groups: one group was NEE and EW, inclined to S and SW. The other group was NW and NWW, inclined to SW and NE. The compressional torsional fault was a reverse fault whose strike was consistent with the axial direction of the anticline or oblique at a small angle (20°–30°) [22]. Wireless sensors were placed in the research object to obtain relevant gas OL data as prediction data.

#### 1.2.4 Experimental index design

According to the above determined experimental objects, this paper took the prediction accuracy and time as the experimental indicators to complete the experimental analysis.

#### 1.2.5 Experimental results

The basic parameters of coal seam gas occurrence were determined through the preprocessing steps of coal mine gas occurrence data based on on-site measurement. And collection in the area are shown in Table 2.

**Table 2:** Basic parameters of coal seam gas occurrence.

Parameter	Unit	Value	Parameter	Unit	Value
Original gas pressure	MPa	1.30	Gas content coefficient	m^3^/t	9.91
Original breathability	M^2^/MPa^2^·d	0.3309	Unit weight of coal	m^3^/t	1.42
Adsorption constant	MPa^−1^	0.223	Moisture content of coal	%	4.81
Porosity of coal	%	6.18	Maximum adsorption capacity	t/m^3^	40.897
Ash content of coal	%	15.06	Original gas content	m^3^/(m^3^·MPa^1/2^)	8.24

The comparison results between the gas content were obtained through multi-source data fusion and the measured production values in the area were shown in Table 3. Where the difference between the gas content were obtained through multi-source data fusion and the actual production measurement value was very small, with an error basically within 4%, and the results obtained have high reliability.

**Table 3:** Comparison results of gas content obtained from multi-source data fusion and actual production measurements in the area.

Name of mining face	Average burial depth(m)	Multi-source data fusion(m^3^/t)	Actual measurement(m^3^/t)	Error(%)	Reliability
2405 North	384	11.02	/	/	Reliable
2403 South	436	03.58	/	/	Reliable
24051(Centre)	511	15.36	14.0	13.2	Reliable
2403 Centre	431	11.24	/	/	Reliable
2403 North	428	16.08	15.5	1.6	Reliable
2305	517	14.7	13.5	1.2	Reliable
2306	520	14.3	14.2	1.2	Reliable
2206 South	513	15.1	14.2	3.6	Reliable
2206 Centre	511	13.4	14.2	−0.8	Reference

**Fig. 4:**
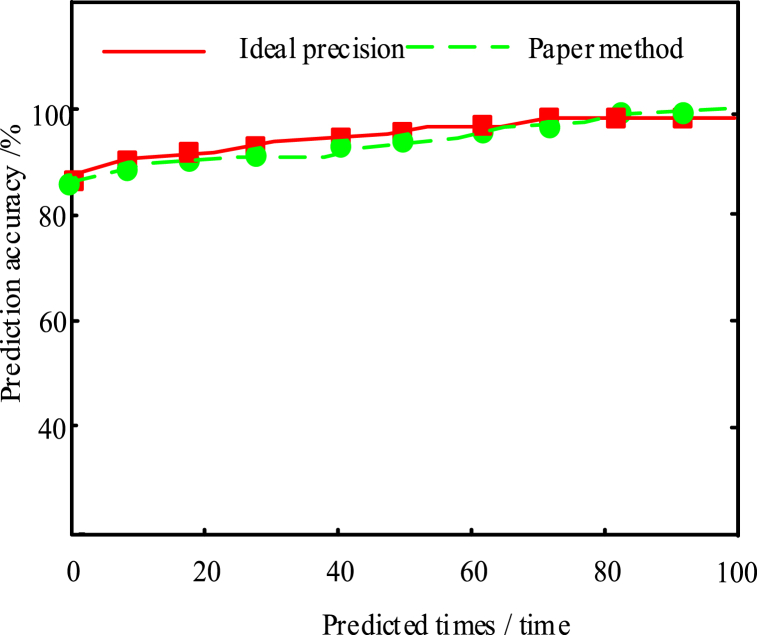
Analysis of the accuracy results of the method prediction in this paper.

To verify the effectiveness of this method, the results of the present method are shown in Fig. 4:

The experimental results in Fig. 4 showed that when predicting the sample objects by using this method, the prediction accuracy was within a reasonable range and was close to the ideal precision, and it was always showing an upward trend and higher than 90%. From the Figure, this method had a certain accuracy.

**Fig. 5:**
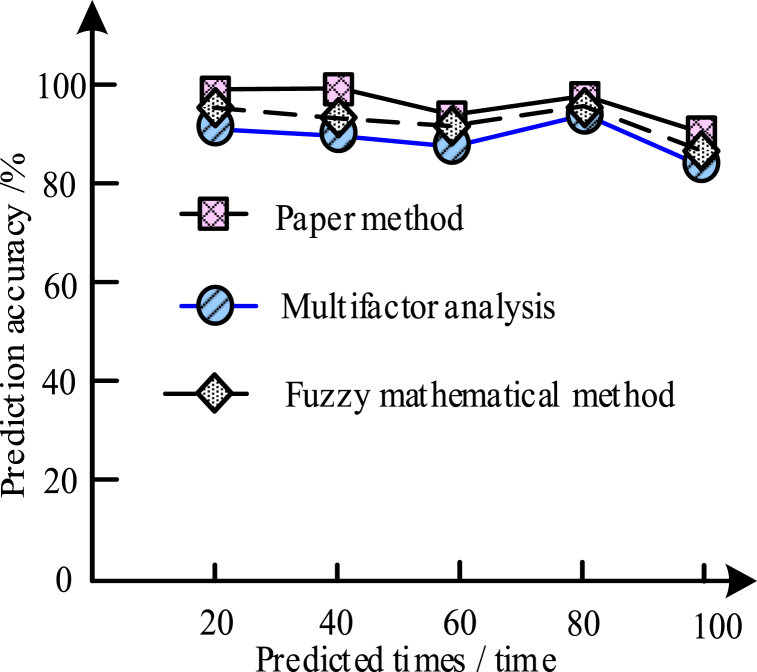
Analysis and comparison of the prediction accuracy between the different methods.

To further verify the accurate determination of this method prediction, the current method, Fuzzy mathematical method and Multifactor analysis were compared in the experiment. The results are shown in Fig. 5:

From the experimental results in Fig. 5, the prediction accuracy comparison using three methods proved that the prediction accuracy of all three methods was relatively high, but the method designed in this paper had some advantages. Among them, the accuracy of this method was up to 98%, while the other two traditional methods were lower than the present method. This was due to the method of this paper placing the sensor in the prediction load object and obtaining response data through the sensor, improving the prediction effect.

**Fig. 6:**
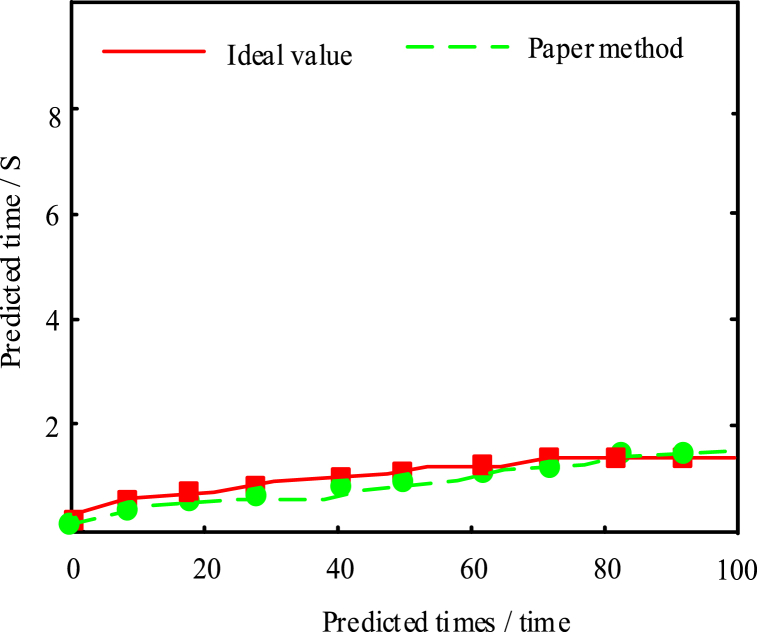
Method prediction time-consuming analysis in this paper.

The predicted time-consuming of this method was also validated in the experiments. The results are shown in Fig. 6:

In Fig. 6, the time consumption of the present method met the actual requirements and was consistent with the actual ideal prediction value. This method not only had the prediction accuracy, but also had a certain work efficiency, and the predicted time consumption was always less than 2s. This was due to the prediction model set by the present method before making the prediction, and preprocessing the predicted data, reducing the interference time of other data, and thus improving the prediction speed of this method.

**Fig. 7:**
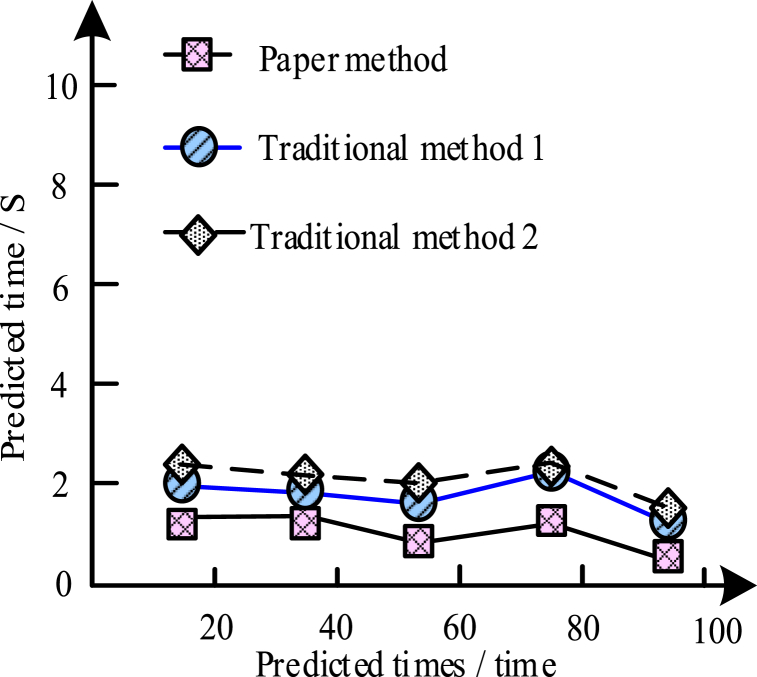
Prediction of the time-consuming analysis by the different methods.

To further verify the time consumption of the current method, traditional methods 1 and 2 were compared in the experiment. They were mining statistics method and grey system method, respectively. The results obtained by the three methods are shown in Fig. 7.

Fig. 7 shows the comparison of time consumption among three prediction methods. From Fig. 7, although the three methods predicted less time-consuming and consistent trends, carefully, this method had the advantage of more speed. This paper prediction method was fast and had certain working efficiency.

## 2 Conclusion

In the prediction method, the paper preprocessed the data to reduce the influence of regular noise data, analyzed the basic principle of multi-source data fusion, and constructed the regular prediction model. It took the weighted factor optimal value as the model input value, and completed the design of coal mine gas law prediction method based on multi-source data fusion. It also verified the effectiveness and feasibility of this method experimentally.

## Funding statement

No funding was received.

## Author contribution statement

Huice Jiao: Conceived and designed the experiments; Wrote the paper.

Weihua Song: Conceived and designed the experiments; Performed the experiments.

Peng Cao: Performed the experiments; Analyzed and interpreted the data.

Dengming Jiao: Analyzed and interpreted the data; Contributed reagents, materials, analysis tools or data.

## Data availability statement

Data will be made available on request.

## Additional information

No additional information is available for this paper.

## Declaration of competing interest

The authors have no interests to declare.

## References

[1] Lyu P., He L., He Z. (2021). Research on remote sensing prospecting technology based on multi-source data fusion in deep-cutting areas. Ore Geol. Rev..

[2] Chang F., Heinemann P.H. (2020). Prediction of human odour assessments based on hedonic tone method using instrument measurements and multi-sensor data fusion integrated neural networks. Biosyst. Eng..

[3] Li J., Wang Y., Nguyen X. (2022). First insights into mineralogy, geochemistry, and isotopic signatures of the Upper Triassic high-sulfur coals from the Thai Nguyen Coal field, NE Vietnam. Int. J. Coal Geol..

[4] Xu Z., Li X., Li J. (2022). Characteristics of source rocks and genetic origins of natural gas in deep formations, Gudian Depression, Songliao Basin, NE China. ACS Earth and Space Chemistry.

[5] Zhan C., Dai Z., Soltanian M.R. (2022). Data-worth analysis for heterogeneous subsurface structure identification with a stochastic deep learning framework. Water Resour. Res..

[6] Liu S.M., Li X.L., Wang D.K. (2020). Investigations on the mechanism of the microstructural evolution of different coal ranks under liquid nitrogen cold soaking. Energy Sources, Part A Recovery, Util. Environ. Eff..

[7] Li X.L., Chen S.J., Liu S.M., Li Z.H. (2021). AE waveform characteristics of rock mass under uniaxial loading based on Hilbert-Huang transform. J. Cent. S. Univ..

[8] Jin X., Wang M., Cui M. (2020). Joint probability density prediction for multiperiod thermal ratings of overhead conductors. IEEE Trans. Power Deliv..

[9] Zheng Z., Zuo Y., Wen H. (2022). Natural gas characteristics and gas-source comparisons of the lower triassic jialingjiang formation, eastern Sichuan basin. J. Petrol. Sci. Eng..

[10] Saini A., Tien I. (2019). Erratum to 'Methodology for real-time prediction of structural seismic risk based on sensor measurements'. Struct. Saf..

[11] Zhou X.M., Wang S., Li X.L. (2022). Research on theory and technology of floor heave control in semicoal rock roadway: taking longhu coal mine in Qitaihe mining area as an Example. Lithosphere.

[12] Wang L., Sun Y., He Y. (2019). Motor health status prediction method based on information from multi-sensor and multi-feature parameters. J. Nondestr. Eval..

[13] Guo W., Wang L., Liu C. (2021). Prediction of gas–liquid two-phase flow rates through a Vertical pipe based on thermal diffusion. Ind. Eng. Chem. Res..

[14] Li X.L., Chen S.J., Wang S. (2021). Study on in situ stress distribution law of the deep mine taking Linyi Mining area as an example. Adv. Mater. Sci. Eng..

[15] Jouybari-Moghaddam Y., Saradjian M.R. (2019). A semi-empirical approach for the estimation of land-surface emissivity from satellite data based on spectral index fusion using ensemble regression. Int. J. Rem. Sens..

[16] Cao W., Dong L., Wu L. (2020). Quantifying urban areas with multi-source data based on percolation theory. Remote Sensing of Environment.

[17] Su M., Liu Y., Xue Y. (2021). Detection method of karst features around tunnel construction by multi-resistivity data-fusion pseudo-3D-imaging based on the PCA approach. Eng. Geol..

[18] Liu H.Y., Zhang B.Y., Li X.L. (2022). Research on roof damage mechanism and control technology of gob-side entry retaining under close distance gob. Eng. Fail. Anal..

[19] Shu B., Zhu R., Zhang S. (2019). A qualitative prediction method of new crack-initiation direction during hydraulic fracturing of pre-cracks based on hyperbolic failure envelope. Appl. Energy.

[20] Curzio D.D., Castrignanò A., Fountas S. (2021). Multi-source data fusion of big spatial-temporal data in soil, geo-engineering and environmental studies. Sci. Total Environ..

[21] Yang Z., Xu J., Feng Q. (2022). Elastoplastic analytical solution for the stress and deformation of the surrounding rock in cold region tunnels considering the influence of the temperature field. Int. J. GeoMech..

[22] Shan Y.F., Gao Z.B. (2020).

